# An automatic Q-factor matching method for eliminating 77% of the ZRO of a MEMS vibratory gyroscope in rate mode

**DOI:** 10.1038/s41378-024-00695-4

**Published:** 2024-05-24

**Authors:** Jingbo Ren, Tong Zhou, Yi Zhou, Yixuan Li, Yan Su

**Affiliations:** School of Mechanical Engineering, Nanjing University of Science and Technology, Nanjing, 210000 China

**Keywords:** Sensors, Electrical and electronic engineering

## Abstract

Mismatching quality factors (Q-factors) is one of the main factors causing zero-rate output (ZRO) in degenerate (DE) Micro-Electro-Mechanical Systems (MEMS) vibratory gyroscopes. To eliminate the ZRO of the DE MEMS gyroscope, this study introduces a method for real-time identification and automatic matching of Q-factors in rate mode. By leveraging the vibration characteristics of the DE MEMS vibratory gyroscope in rate mode, dedicated online test methods are designed to determine the Q-factors for both the drive and sense modes, enabling online identification of the Q-factor mismatching. Furthermore, an automatic Q-factor matching system is designed utilizing the mechanical-thermal dissipation mechanism of the resistive damper. The effectiveness of this proposed method is validated through simulations and experiments conducted on a MEMS disk resonator gyroscope (DRG). The results show a measurement error within 4% for Q-factor identification, and automatic Q-factor matching effectively reduces the ZRO by 77%. Employing this automatic Q-factor matching method successfully reduces the ZRO that is caused by the mismatching of Q-factors in the MEMS DRG from 0.11°/s to 0.025°/s and improves the bias instability (BI) from 0.40°/s to 0.19°/s.

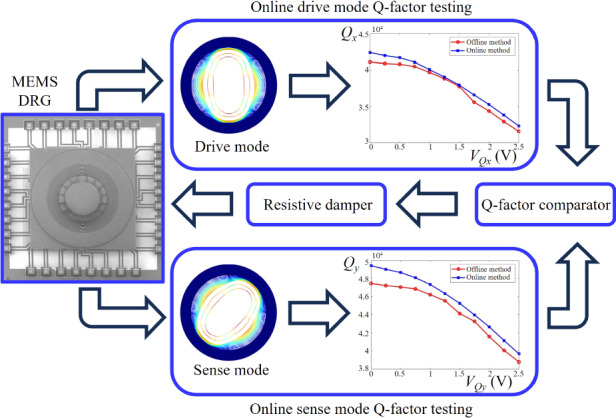

## Introduction

Microelectromechanical system (MEMS) vibratory gyroscopes are micromachined inertial sensors that are utilized for measuring the rate or angle of rotation^1,2^. These devices are extensively applied in the aerospace industry, automobiles, smartphones, game controllers, and various other fields, enabling precise positioning, navigation, and motion control^3^. Their notable advantages include their small size, low power consumption, and swift response. MEMS vibratory gyroscopes operate based on the principles of the Coriolis effect and can be categorized into two types according to their operation mode: Type I and Type II gyroscopes^4^. In the past few years, Type II MEMS vibratory gyroscopes have achieved remarkable performance. Challoner et al. reported a disc resonator gyroscope with an angle random walk (ARW) of 0.0035°/√h and a bias instability of 0.012°/h. The gyroscope operates under mode-matching conditions, with a resonator diameter of 8 mm and an overall structural volume of approximately 6.4 mm^3^, enclosed in a leaded chip carrier (LCC) vacuum package^5^. Endean et al. proposed a tuning fork MEMS gyroscope operating with frequency separation and a bandwidth greater than 700 Hz. The ARW of the gyroscope is less than 0.006°/√h, and the median bias stability under temperature variation is 0.2°/h^6^. Koenig et al. provided an overview of the latest advancements made by Northrop Grumman Corporation in the field of high-performance MEMS gyroscopes. The randomly selected gyroscopes exhibited an average ARW of less than 0.021°/√h and an average BI of less than 0.016°/h. The best-performing device even achieved a BI as low as 0.007°/h^7^. Gadola et al. reported a novel gyroscope based on nanoresistive sensing, with a sensor footprint size of 1.3 mm^2^ and an overall silicon structure volume of 0.026 mm^3^. The ARW of the gyroscope can reach 0.0042°/√h, and the BI at 1000 s is 0.014°/h^8^. These achievements represent state-of-the-art performances that MEMS gyroscopes can currently achieve in the rate mode, providing more possibilities for navigation-grade applications of MEMS gyroscopes.

Type II MEMS vibratory gyroscopes operate in the rate mode for measuring the rotational angular rate. The drive mode is typically excited by an electrostatic driving force to achieve a resonant state with constant amplitude, while the sense mode is excited by the Coriolis force induced by rotation. Both the drive mode and the sense mode can be degenerate (DE) or nondegenerate (NDE)^9,10^. The zero-rate output (ZRO) is one of the important factors determining the performance of a MEMS gyroscope. In general, the main causes of ZRO are quadrature leakage^11^, synchronous demodulation phase error^12^, parasitic feed-through capacitance^13^, and temperature variation^14^. With the introduction of techniques such as quadrature error suppression^15^, phase compensation^16^, electromechanical amplitude modulation^17^, and temperature compensation^18^, the abovementioned problems have been properly addressed. Thus, the in-phase error caused by the damping asymmetry gradually has become the main factor leading to ZRO, especially for DE MEMS vibratory gyroscopes. This occurs because for MEMS gyroscopes with identical drive and sense modes, it is essential that their resonant structures possess impeccable symmetry^19^. Unfortunately, due to manufacturing imperfections, the resonant structure of a fabricated gyroscope is not perfectly symmetrical due to the nonuniform distribution of crucial structural parameters such as stiffness and damping. Consequently, the primary sources of errors that impact gyroscope performance stem from the asymmetry in stiffness and damping. For MEMS gyroscopes packaged at the wafer level with high vacuum, thermoelastic damping constitutes the main component of the gyroscope’s structural damping, and thermoelastic damping is highly sensitive to temperature^20^. Therefore, when there are significant fluctuations in the external temperature, the damping error also fluctuates with temperature, resulting in ZRO drift.

Damping is the primary form of energy dissipation in MEMS gyroscopes, and the Q-factor serves as a quantitative indicator of this energy dissipation. Thus, the damping asymmetry can be transformed into a Q-factor mismatching problem for MEMS gyroscopes under certain circumstances. Mechanical tuning and electrical tuning are currently the mainstream methods for Q-factor matching. Hamza et al. proposed a Q-factor matching method for ring-shaped MEMS gyroscopes, which utilized the thermally induced energy dissipation effect to effectively control the Q-factor difference between gyroscope modes within 2.32%^21^. Jandakl et al. reported a method of depositing a 30 nm platinum film selectively on different parts of a MEMS gyroscope through sputtering to change the damping coefficient of the gyroscope in the nonoperational mode. The experimental results showed that this method effectively altered the Q-factor of the MEMS gyroscope^22^. Although the aforementioned mechanical tuning methods can achieve Q-factor matching, the results are usually irreversible, thus greatly limiting the application environment of such methods.

In contrast, a method for electrically tuning the Q-factor is characterized by low cost, high flexibility, and the potential for dynamic matching, and thus has been used in widespread applications. Tsukamoto et al. reported an automatic compensation method for the frequency and Q-factor of a rate-integrating gyroscope (RIG), which compensates for mismatches by measuring and compensating for the cross-coupling terms between two different vibration modes, reducing the compensated cross-coupling terms by three orders of magnitude^23^. Chen et al. utilized the electrostatic softening effect to tune the Q-factor of a gyroscope by enhancing squeeze-film damping and reducing mode coupling within a 2-axis dual mass resonator through the application of bias voltage, achieving a tuning range of 21%^24^. Moreover, they proposed a similar electrostatic softening-based Q-factor matching method for a three-mass MEMS resonator composed of two outer masses and one inner mass, in which the Q-factor of the resonator was adjusted by increasing the anchor damping via applied bias voltage, resulting in a maximum decrease of 64.8% in the resonator’s Q-factor^25^.

In addition to the Q-factor tuning method based on the electrostatic softening effect, the Q-factor tuning method of resistive damping based on the mechanical-thermal dissipation mechanism is also being continuously developed and applied. This resistive damping model was originally proposed by Duwel et al.^26^, who discussed the impact of the resistive damping caused by the input impedance of the external amplifier on the Q-factor of MEMS gyroscopes. Tatar et al. successfully adjusted the Q-factor of a mode-matched MEMS gyroscope by using resistors and an external DC potential^27^. Deimerly et al. reported a method of coupling a microresonator to a resistor that can provide additional damping sources to control the mechanical damping of MEMS devices. By tuning the Q-factor, this method provides an effective solution to the cointegration problem between accelerometers and gyroscopes in the same MEMS cavity under low pressure^28^. Fain et al. compared and verified the suppression effect of squeeze-film damping and electromechanical damping on M&NEMS accelerometers at 1 mbar through experiments, and they proposed that the electromechanical damping technique seems to be more promising for addressing the cointegration problem of accelerometers and gyroscopes in high-vacuum packaging conditions^29^. Gando et al. introduced an adjustable resistive damper for MEMS gyroscopes based on comb-like capacitive sensitive structures. By adjusting the resistance and DC voltage, this resistive damper achieved fine-tuning of the Q-factor of MEMS gyroscopes^30^. Guo et al. further extended the theory of resistance-damping adjustment specifically for flat plate capacitive sensitive structures. In the whole-angle mode, this theory reduced the asymmetry error in the gyroscope’s damping by 87%^31^.

In conclusion, the current methods for electrically tuning the Q-factor of MEMS gyroscopes can be summarized as follows: reducing mode coupling, leveraging the electrostatic softening effect of bias voltage, and utilizing resistive damping techniques based on the mechanical-thermal dissipation mechanism. However, these methods often necessitate additional test procedures to ascertain gyroscope Q-factors or attenuation times, and using them to meet dynamic Q-factor matching requirements can prove challenging. Moreover, most of these methods involve open-loop Q-factor matching with low efficiency.

This study considers the ZRO problem caused by Q-factor mismatches when operating in rate mode. To improve the performance of DE MEMS gyroscopes in rate mode, this research focuses on MEMS DRGs, and a real-time identification and automatic matching method for the Q-factor in rate mode is proposed. Based on the vibration characteristics of MEMS DRGs in rate mode, online test methods for the Q-factors of the drive and sense modes are independently designed. By utilizing the damping adjustment capability of a resistive damper, an automatic Q-factor matching system is designed to output the Q-factor matching voltage that eliminates the Q-factor mismatches of the MEMS DRG, thereby reducing the ZRO and improving gyroscope performance. The corresponding results have important implications for other DE MEMS gyroscopes in rate mode.

## Results

### Structural design of the MEMS DRG

**Fig. 1:**
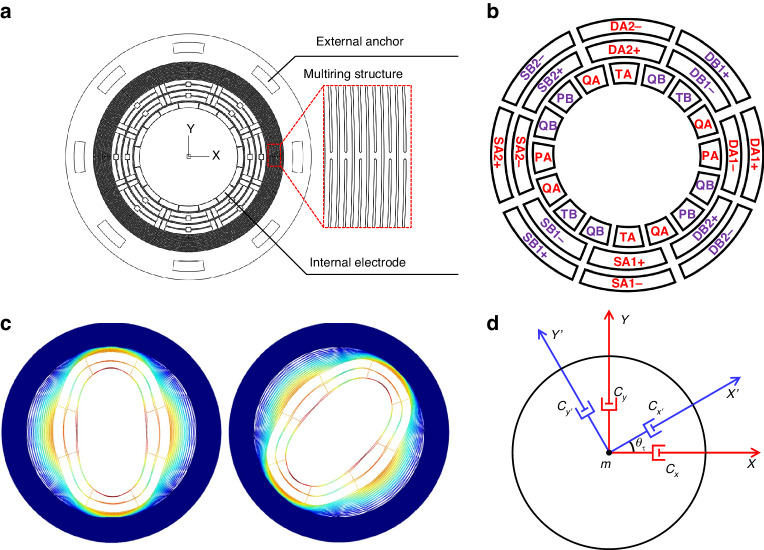
External anchoring structure and asymmetric damping in the MEMS DRG. **a** Mechanical structure of the MEMS DRG. **b** Configuration of internal electrodes in the MEMS DRG. **c** Vibration shape of the MEMS DRG mode. **d** Damping asymmetry error model of the MEMS DRG

The proposed method for the online identification and automatic matching of Q-factor matching in rate mode is applied to a MEMS DRG with fixed external anchors. Figure 1a depicts the mechanical structure of the MEMS DRG, which consists of external anchors, a multiring resonant structure, and internal electrodes. The external anchors are uniformly distributed on the outermost side of the multiring resonant structure, which is securely anchored to the silicon substrate. The multiring resonant structure is interconnected by concentric rings of increasing radius through spokes, while the internal electrodes are positioned at the innermost side of the multiring resonant structure to enable control over the MEMS DRG. Figure 1b shows a schematic diagram depicting the specific configuration of the internal electrodes in the MEMS DRG. A total of 32 discrete electrodes were employed, with 16 electrodes dedicated to both drive and sense modes. The drive mode is motivated by the driving electrodes (DB1 and DB2), while the sensing electrodes (SB1 and SB2) detect the displacement vibration of the drive mode. The quadrature error suppression electrodes (QBs) enable adjustment of the stiffness deflection angle in drive mode, while the tuning electrodes (TBs) allow for resonant frequency adjustments. Q-factor matching electrodes (PBs) are used to fine-tune the damping coefficient in drive mode. The electrode configuration remains consistent between the drive and sense modes.

The MEMS DRG exhibits degenerate drive and sense modes, in which the vibration of the drive mode induces vibrations in the sense mode through the Coriolis effect. Consequently, the sense mode includes vibrations that enable the measurement of the external rotation angular rate or angle by detecting the vibration displacement. Figure 1c depits the mode shapes of the MEMS DRG with external anchors. In comparison to a MEMS DRG with central anchors, the vibration energy of a MEMS DRG with external anchors is concentrated within the innermost ring of the multiring structure, resulting in greater vibration displacement inside the structure. This configuration prevents dissipation of vibration energy toward the outer ring and minimizes the conduction of external stress toward the inner ring. As a result, it effectively enhances both the Q-factor and the conversion coefficient of the Coriolis force.

### Q-factor matching analysis of the MEMS DRG

Figure 1d shows the model depicting the asymmetry in damping experienced by the MEMS DRG. The vibration directions of the drive and sense modes correspond to the X-axis and Y-axis of the vibration coordinates (X-Y), respectively. The damping along the X-axis in the drive mode is denoted as *c*_*x*_, and the damping along the Y-axis in the sense mode is denoted as *c*_*y*_. The damping coordinates (X′-Y′) align with the damping axis of the MEMS DRG, with *c*_*x*′_ representing the damping along the X′-axis in drive mode and *c*_*y*′_ representing the damping along the Y′-axis in sense mode. *θ*_*τ*_ represents the angle between the damping coordinates and vibration coordinates, also referred to as the damping deflection angle. The presence of *θ*_*τ*_ results in energy coupling between both operational modes, leading to an in-phase error with respect to the Coriolis force. Consequently, separating the in-phase error from the Coriolis force during the angular rate demodulation process becomes challenging.

By utilizing the projection method to transform the coordinates of X′-Y′ and X-Y, the damping matrix *C* in the vibration coordinates can be derived as follows:

1 $$\begin{array}{l}C=\left[\begin{array}{cc}{c}_{x} & {c}_{xy}\\ {c}_{xy} & {c}_{y}\end{array}\right]\\\quad =\left[\begin{array}{cc}\frac{{c}_{x\text{'}}+{c}_{y\text{'}}}{2}+\frac{{c}_{x\text{'}}-{c}_{y\text{'}}}{2}\,\cos 2{\theta }_{\tau } & \frac{{c}_{x\text{'}}-{c}_{y\text{'}}}{2}\,\sin 2{\theta }_{\tau }\\ \frac{{c}_{x\text{'}}-{c}_{y\text{'}}}{2}\,\sin 2{\theta }_{\tau } & \frac{{c}_{x\text{'}}+{c}_{y\text{'}}}{2}-\frac{{c}_{x\text{'}}-{c}_{y\text{'}}}{2}\,\cos 2{\theta }_{\tau }\end{array}\right]\end{array}$$

where *c*_*xy*_ represents the damping coupling coefficient between the gyroscope modes, yielding the emergence of the damping mismatch force *F*_*cxy*_, which is coupled from the drive mode to the sense mode as ${c}_{xy}\dot{x}$.

Moreover, the closed-loop scale factor *SF* of the MEMS DRG in rate mode can be expressed as follows:

2 $$SF=\frac{2m{A}_{g}\dot{x}\Omega }{\Omega }=2m{A}_{g}{\omega }_{x}|x|$$

where *m* denotes the equivalent mass of the MEMS DRG, *A*_*g*_ represents the angular gain coefficient, *ω*_*x*_ symbolizes the resonant frequency of the drive mode, *x* signifies the vibration displacement of the drive mode, and Ω indicates the external angular rate input. Thus, the ZRO induced by Q-factor mismatches can be elegantly expressed as follows:

3 $${\Omega }_{ZRO}=\frac{{c}_{xy}}{2m{A}_{g}}=\frac{\tan 2{\theta }_{\tau }}{4{A}_{g}}\left(\frac{{\omega }_{x}}{{Q}_{x}}-\frac{{\omega }_{y}}{{Q}_{y}}\right)$$

where ${Q}_{x}=m{\omega }_{x}/{c}_{x}$ and ${Q}_{y}=m{\omega }_{y}/{c}_{y}$ denote the Q-factors of the drive mode and sense mode, respectively. When the gyroscope is in the mode-matching state, ${\omega }_{x}={\omega }_{y}$. Equation (3) can ultimately be simplified as follows:

4 $${\Omega }_{ZRO}=\frac{{\omega }_{x}\cdot \,\tan 2{\theta }_{\tau }}{4{A}_{g}}\left(\frac{{Q}_{y}-{Q}_{x}}{{Q}_{x}{Q}_{y}}\right)$$

According to (4), the ZRO resulting from the Q-factor mismatches exhibits a direct correlation with *ω*_*x*_, *A*_*g*_, *θ*_*τ*_, *Q*_*x*_, and *Q*_*y*_. By ensuring an aligned adjustment of the Q-factors for both the drive mode and sense mode, it is feasible to entirely eradicate the ZRO, thereby enhancing the performance of the MEMS DRG.

### Control scheme of the Q-factor matching method

**Fig. 2:**
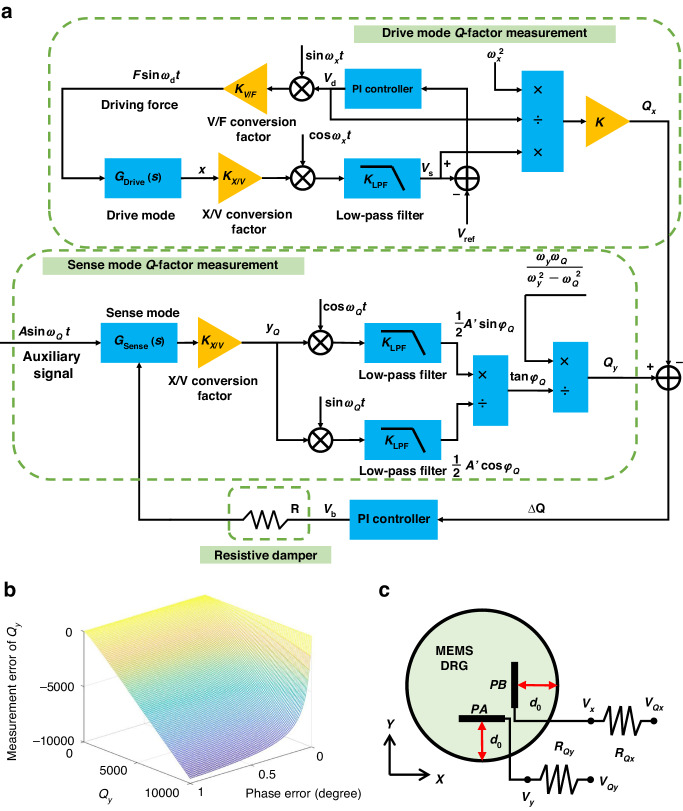
Schematic diagram of the automatic Q-factor matching system and analysis of measurement errors. **a** Control scheme for real-time automatic identification and matching of the Q-factor. **b** Measurement error of Qy with respect to the phase error φe and Qy. **c** Resistive damper model of the MEMS DRG

Figure 2a depicts the control scheme proposed in this research for the real-time automatic identification and matching of the Q-factor. This scheme consists of three key components: the drive mode Q-factor online test section, the sense mode Q-factor online test section, and the resistive damper section. Based on the analysis of the Q-factor mismatches in the MEMS DRG, the discrepancy between the Q-factors of the drive mode and the sense mode serves as a metric signal for the Q-factor mismatches. Aligning the Q-factors between gyroscope modes enables effective Q-factor matching. The drive mode Q-factor online test method determines the drive mode Q-factor by analyzing the amplitude of the gyroscope driving displacement. Furthermore, an auxiliary signal is introduced to excite the sense mode via its driving electrode, enabling the decomposition of the phase of the response signal generated by this auxiliary signal after affecting the sense mode. By subtracting the obtained *Q*_*x*_ and *Q*_*y*_ values, online identification of the Q-factor mismatches can be accomplished. Finally, leveraging the difference in Q-factors between modes as a controlled parameter, adjustments are made to the damping coefficient along the gyroscope vibration axis using a resistive damper. The PI controller automatically generates a Q-factor matching voltage to produce a zero difference in the Q-factors between gyroscope modes, thereby achieving automatic Q-factor matching in both the drive and sense modes and eliminating the ZRO of the MEMS DRG.

### Drive mode Q-factor online test method

As depicted in Fig. 2a, the driving force acting on the drive mode *G*_*Drive*_(*s*) is defined as ${F}_{x}=F\,\sin ({\omega }_{x}t)$, where *F* represents the magnitude of *F*_*x*_. The resulting vibration displacement *x* induced by the drive mode can be mathematically formulated as follows:

5 $$x(t)=\frac{F{Q}_{x}}{m{\omega }_{x}^{2}}\,\cos ({\omega }_{x}t)$$

According to (5), following the stimulation of the drive mode, the magnitude of *x* produced by the drive mode exhibits a correlation with the Q-factor. Consequently, at this juncture, the low-pass filtered output signal *V*_*s*_ and the output signal *V*_*d*_ from the PI controller are expressed as follows:

6 $${V}_{s}=\frac{1}{2}\frac{F{Q}_{x}{K}_{X/V}{K}_{LPF}}{m{\omega }_{x}^{2}}$$

7 $${V}_{d}=\frac{F}{{K}_{V/F}}$$

where *K*_*X/V*_ symbolizes the displacement-to-voltage conversion factor, *K*_*LPF*_ represents the filter gain, and *K*_*V/F*_ signifies the voltage-to-electrostatic force conversion factor. *Q*_*x*_ can be expressed as follows:

8 $${Q}_{x}=2\left(\frac{m}{{K}_{X/V}{K}_{LPF}{K}_{V/F}}\right)\frac{{\omega }_{x}^{2}{V}_{s}}{{V}_{d}}=K\frac{{\omega }_{x}^{2}{V}_{s}}{{V}_{d}}$$

where *K* is defined as the electromechanical interface parameter of the MEMS DRG. By combining *K*_*X/V*_, *K*_*LPF*_, *K*_*V/F*_, and the relevant structural parameters of the MEMS DRG, the theoretical value of *K* can be calculated as 9.7 × 10^–8^ s^2^. Compared to the experimentally obtained calibrated value of *K*, the relative error is less than 2%. *K* exhibits a negative temperature coefficient and decreases with increasing in ambient temperature. Within the temperature range of –40 °C to 60 °C, the relative change in *K* is 5%. Therefore, under stable environmental conditions, this value can be considered constant.

The parameters *ω*_*x*_, *V*_*s*_, and *V*_*d*_ can be continuously monitored in the circuit. By performing algebraic operations on these parameters, *Q*_*x*_ can be derived.

### Sense mode Q-factor online test method

As depicted in Fig. 2a, an ancillary signal *V*_*Q*_(*t*) = *A*sin(*ω*_*Q*_*t*) is imparted onto the driving electrode of the sense mode *G*_*Sense*_(*s*). Here, *A* denotes the magnitude of the ancillary signal, while *ω*_*Q*_ signifies its frequency. The resultant output *y*_*Q*_ of the ancillary signal subsequent to the sense mode can be expressed as follows:

9 $$\begin{array}{l}{y}_{Q}(t)={A}_{Q}\,\sin ({\omega }_{Q}t+{\varphi }_{Q})\\\quad {A}_{Q}=\frac{A{K}_{C/V}}{m\sqrt{{({\omega }_{y}^{2}-{\omega }_{Q}^{2})}^{2}\,+\,{(\frac{{\omega }_{y}{\omega }_{Q}}{{Q}_{y}})}^{2}}}\\\quad\, {\varphi }_{Q}=\arctan \frac{{\omega }_{y}{\omega }_{Q}/{Q}_{y}}{{\omega }_{y}^{2}-{\omega }_{Q}^{2}}\end{array}$$

where *A*_*Q*_ and *φ*_*Q*_ are the amplitude and phase of *y*_*Q*_, respectively.

Equation (9) demonstrates that, in the absence of considering the uncertain phase error introduced by the hardware circuitry, the phase *φ*_*Q*_ solely depends on the frequency *ω*_*Q*_ of the ancillary signal, as well as the frequency *ω*_*y*_ and Q-factor *Q*_*y*_ of the sense mode. Because the sense mode operates with forced vibration in rate mode, its frequency *ω*_*y*_ cannot be directly measured. Thus, it becomes imperative to employ a real-time automated mode-matching technique to finely tune the gyroscope frequencies such that both the drive and sense modes exhibit harmonious congruence (*ω*_*x*_ = *ω*_*y*_). By employing sin(*ω*_*Q*_*t*) and cos(*ω*_*Q*_*t*) as demodulation reference signals, orthogonal demodulation and in-phase demodulation are separately performed on *y*_*Q*_, yielding the orthogonal components *A*_1_ and in-phase components *A*_2_, as follows:

10 $${A}_{1}=LPF\{{y}_{Q}\cdot \,\cos {\omega }_{Q}t\}=\frac{1}{2}A{^{\prime}}\,\sin {\varphi }_{Q}$$

11 $${A}_{2}=LPF\{{y}_{Q}\cdot \,\sin {\omega }_{Q}t\}=\frac{1}{2}A{^{\prime}}\,\cos {\varphi }_{Q}$$

Eventually, the expression for *Q*_*y*_ in the sense mode can be derived from the aforementioned equations, which can be represented as follows:

12 $${Q}_{y}=\frac{{\omega }_{y}{\omega }_{Q}}{{\omega }_{y}^{2}-{\omega }_{Q}^{2}}\cdot \frac{{A}_{2}}{{A}_{1}}$$

However, in practical situations, phase errors caused by hardware circuits are inevitable and have a negative impact on the online testing of *Q*_*y*_. By substituting in the true parameters of the MEMS DRG, we obtain a curve depicting the variation in the measurement error of *Q*_*y*_ with respect to the phase errors *φ*_*e*_ and *Q*_*y*_, as shown in Fig. 2b. The larger the phase error *φ*_*e*_ is, the greater the measurement error of *Q*_*y*_. Moreover, if the Q-factor of the gyroscope is larger, then the measurement error of the Q-factor caused by the phase error will also be larger. In other words, when applying the Q-factor test method to MEMS gyroscopes with high Q-factors, a very small phase error is required to ensure a sufficiently small measurement error.

To reduce the measurement error of *Q*_*y*_ and minimize the influence of the phase error *φ*_*e*_ on other control loops, we measure and compensate for the phase error in the designed hardware circuit from the very beginning and control *φ*_*e*_ within 0.1°. In this scenario, the theoretical measurement error of *Q*_*y*_ is less than 2%.

### Principle of the resistive damper

To enable adjustable damping for the MEMS DRG, we incorporate resistive dampers that consist of resistance and voltage into the vibration direction of both the drive and sense modes. Plate capacitors are established between the multiring structure and the Q-factor matching electrodes (PAs and PBs). During vibration, a current is induced, resulting in heat dissipation as the current flows through the resistance. By converting mechanical energy into heat dissipation, the damping magnitude can be effectively regulated along the mode’s vibration direction. The resistive damper model of the MEMS DRG is shown in Fig. 2c.

Considering the resistive damper as the subject of analysis in the drive mode vibration direction, the influence of the resistive damper on the motion equation of the drive mode can be expressed as follows:

13 $$m\ddot{x}+{c}_{x}\dot{x}+{k}_{x}x=\frac{1}{2}\frac{\partial }{\partial x}[C(x){V}_{x}^{2}]$$

where *C*(*x*) signifies the capacitance of the plate capacitor, *V*_*Qx*_ denotes the Q-factor matching voltage exerted on the resistance *R*_*Qx*_, and *V*_*x*_ denotes the effective voltage applied to the PBs. According to Ohm’s law, by utilizing the voltage *V*_*Qx*_ across the Q-factor matching electrode and the resistance *R*_*Qx*_, the actual voltage *V*_*x*_ applied to the Q-factor matching electrode can be calculated:

14 $$\begin{array}{l}{V}_{x}={V}_{Qx}-{R}_{Qx}\frac{d(C(x){V}_{x})}{dt}\\\quad\;\, ={V}_{Qx}-{R}_{Qx}{V}_{x}\frac{d(C(x))}{dt}-{R}_{Qx}C(x)\frac{d({V}_{x})}{dt}\\\quad\;\,={V}_{Qx}-{R}_{Qx}{V}_{x}\frac{d(C(x))}{dt}+{R}_{Qx}^{2}C(x)\frac{{d}^{2}(C(x){V}_{x})}{{d}^{2}t}\end{array}$$

Based on the comparison between the time constant τ = *R*_*Qx*_*C*_0_ and the frequency *ω*_*x*_, if *R*_*Qx*_*C*_0_<<2π/*ω*_*x*_, then the influence of the third term on the right side of (14) can be ignored. Disregarding the higher-order components linked to the resistor *R*_*Qx*_, the expression of *V*_*x*_ can be streamlined as follows:

15 $${V}_{x}={V}_{Qx}/1-\frac{{R}_{Qx}{C}_{0}/{d}_{0}}{{({d}_{0}+x)}^{2}}\dot{x}$$

where *C*_0_ represents the initial capacitance of *C*(*x*) and *d*_0_ signifies the initial spacing of *C*(*x*). By merging (13) and (15), the dynamic equation for the drive mode can be derived as follows:

16 $$m\ddot{x}+{c}_{x}\dot{x}+{k}_{x}x\approx -\frac{{V}_{Qx}^{2}}{2}\left(\frac{{C}_{0}}{{d}_{0}}\right)-3{R}_{Qx}{V}_{Qx}^{2}{\left(\frac{{C}_{0}}{{d}_{0}}\right)}^{2}\dot{x}+\frac{{V}_{Qx}^{2}}{{d}_{0}}\left(\frac{{C}_{0}}{{d}_{0}}\right)x$$

According to (15), *R*_*Qx*_ influences the damping of the drive mode, while *V*_*Qx*_ affects both the damping and stiffness of the drive mode. The damping Δ*c* induced by the resistive damper is positive, whereas the stiffness Δ*k* is negative ($\Delta c=3{R}_{Qx}{V}_{Qx}^{2}{({C}_{0}/{d}_{0})}^{2}$, $\Delta k=-({C}_{0}/{d}_{0}^{2}){V}_{Qx}^{2}$).

The magnitude of Δ*c* increases with increasing of *R*_*Qx*_ and *V*_*Qx*_. However, it is important to note that for the smooth flow of current through the resistance in a plate capacitor, it is necessary to satisfy *R*_*Qx*_*C*_0_<<2π/*ω*_*x*_. Moreover, the issue of stiffness alteration due to resistive dampers is addressed through the implementation of automatic mode-matching technology. This technology serves two purposes in this research: online Q-factor testing and resolving frequency mismatching caused by *V*_*Qx*_.

### Q-factor matching system simulation model

**Fig. 3:**
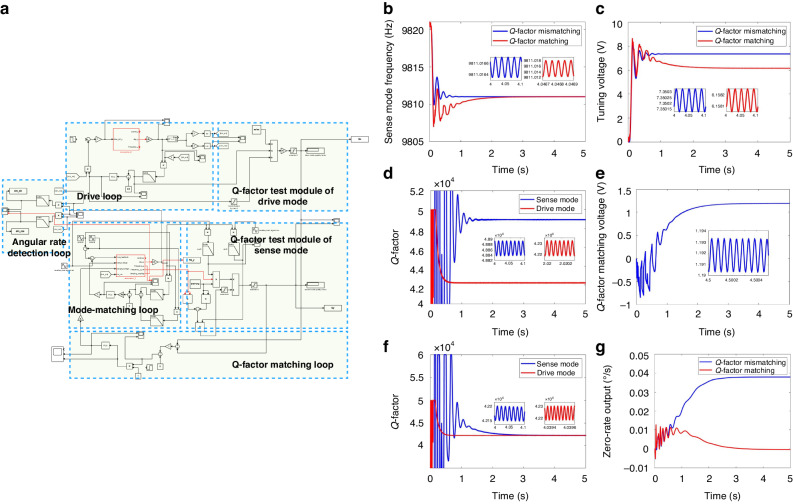
Simulation results of the automatic Q-factor matching method. **a** Simulation model of the automatic Q-factor matching system. **b** Sense mode frequency. **c** Tuning voltage. **d** Q-factor before matching. **e** Q-factor matching voltage. **f** Q-factor after matching. **g** Zero-rate output

To verify the efficacy of the proposed online identification and automatic matching method for the Q-factor, Simulink software is utilized to construct a simulation model of an automatic Q-factor matching system. The control block diagram depicted in Fig. 2a serves as the basis for this model, as shown in Fig. 3a. For simplicity, discussion of the suppression process of the quadrature error signal is omitted in this simulation. Consequently, the model primarily encompasses the drive loop, mode-matching loop, Q-factor matching loop, and angular rate detection loop. The drive mode is stimulated by Simulink’s phase-locked loop (PLL) module and automatic gain control (AGC) module. The PLL module generates the reference signals employed within the simulation model. The mode-matching loop tunes the frequencies of both drive and sense modes, facilitating Q-factor testing as well as addressing the coupling issue arising from the resistive damper operations affecting damping and stiffness. The Q-factor matching loop incorporates Q-factor test modules for both the drive and sense modes alongside the resistive damper. These modules acquire Q-factor mismatching information that is subsequently nullified upon applying the resistive damper, thus achieving automatic compensation for the Q-factor mismatches. Finally, the angular rate detection loop facilitates angular rate detection within the system. Table 1 outlines the key simulation parameters employed in our model.

**Table 1:** Parameters of the simulation model

Parameter	Value	Unit
Resonant frequency of the drive mode *ω*_*x*_	9811	Hz
Resonant frequency of the sense mode *ω*_*y*_	9821	Hz
Damping coefficient of the drive mode *c*_*x*_	1.202 × 10^–7^	N/(m/s)
Damping coefficient of the drive mode *c*_*y*_	1.042 × 10^–7^	N/(m/s)
Effective mass *m*	5.034 × 10^–7^	kg
Angular gain factor *A*_*g*_	0.8	
Displacement to voltage conversion factor *K*_*X/V*_	3.2 × 10^6^	V/m
Voltage to force conversion factor *K*_*V/F*_	3.2 × 10^–6^	N/V

The entire simulation model is executed for a duration of 5 s. Following the operation of the mode-matching loop, Fig. 3b, c shows the sense mode frequency and the tuning voltage. The tuning voltage ultimately stabilizes at 7.35 V before Q-factor matching, while the sense mode frequency decreases from 9821 Hz to 9811.017 Hz. The tuning precision of the mode-matching loop is measured at 0.017 Hz, which falls within the mechanical bandwidth of the gyroscope, thus confirming its mode matching at this juncture. Figure 3d shows the Q-factor test results for both the drive mode and the sense mode, revealing values of 42,221 and 48,872, respectively. The Q-factors obtained by the attenuation method are compared with those obtained by the online test simulation model. The measurement errors of *Q*_*x*_ and *Q*_*y*_ are no greater than 3%, as shown in Table 2.

**Table 2:** Q-factor test results

Test method	*Q*_*x*_	*Q*_*y*_	Δ*Q*	measurement error of *Q*_*x*_ and *Q*_*y*_ (%)
Attenuation method	41,096	47,467	6371	
Simulation results of online method	42,221	48,872	6651	3
Experimental results of online method	42,374	49,147	6773	4

To achieve automatic Q-factor matching, it is imperative to modulate the damping coefficient of the gyroscope through a resistive damper. Optimal control of the Q-factor matching voltage within the model is recommended to fall between 0 V and 5 V, remaining lower than the tuning voltage. Numerical analysis revealed that for the damping coefficient of the MEMS DRG, the resistance *R*_*Qy*_ of the resistive damper needs to be fixed between 10 kΩ and 100 kΩ. Therefore, in this simulation, *R*_*Qy*_ is set at 50 kΩ. With the influence of the resistive damper upon the mode-matching loop, the tuning voltage gradually settles at 6.16 V following a state of Q-factor matching. However, the sense mode frequency remains relatively constant both before and after the process of Q-factor matching since the resistive damper plays a role in frequency tuning. Figure 3e, f depicts the Q-factor matching voltage and Q-factors for both the drive and sense modes. After stability is reached within the Q-factor matching loop, the Q-factor matching voltage settles at 1.19 V, with the Q-factor of the drive mode remaining at 42,221, while that of the sense mode adjusts to 42,226. The MEMS DRG’s Q-factor mismatches decreased by 99%. Figure 3g displays the ZRO of the angular rate detection loop before and after Q-factor matching. The ZRO induced by the Q-factor mismatches decreases from 3.8 × 10^–2^°/s to –3.6 × 10^–4^°/s, resulting in a reduction of two orders of magnitude.

### Implementation of the Q-factor matching circuit

**Fig. 4:**
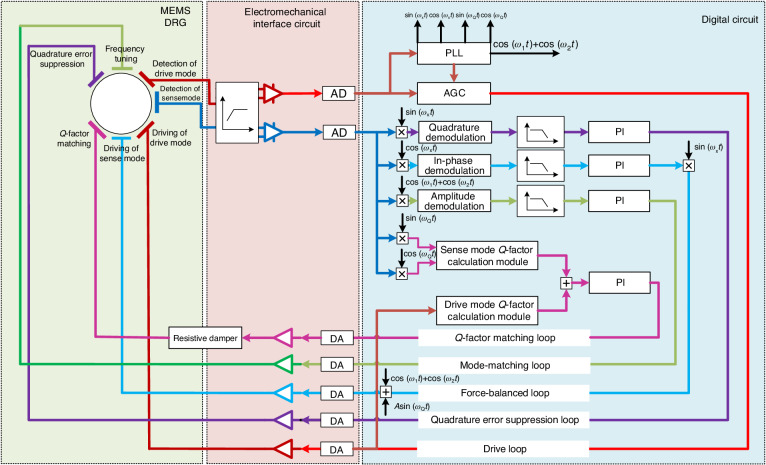
Comprehensive framework of the measurement and control circuit integrating automatic Q-factor matching

Figure 4 shows the comprehensive framework of the measurement and control circuit integrating automatic Q-factor matching. The entire circuit can be partitioned into three key components: the MEMS DRG, an electromechanical interface circuit, and a digital control circuit with a field programmable gate array (FPGA). The digital control circuit comprises five control loops: the drive loop, quadrature error suppression loop, mode-matching loop, Q-factor matching loop, and force-balanced loop.

The drive loop encompasses the AGC and the PLL, which ensures that the drive mode maintains a constant amplitude resonant state while providing reference signals for modulation and demodulation processes in other control loops. The quadrature error suppression loop serves to mitigate the quadrature error signal induced from the drive mode to the sense mode. The mode-matching loop facilitates frequency tuning between gyroscope modes, facilitating online Q-factor testing and addressing stiffness coupling issues arising from the resistive damper. The Q-factor matching loop enables Q-factor matching among gyroscope modes by compensating for Q-factor mismatches through online Q-factor testing and the adjustment capabilities of the resistive damper. Finally, the force-balanced loop is responsible for angular rate detection.

**Fig. 5:**
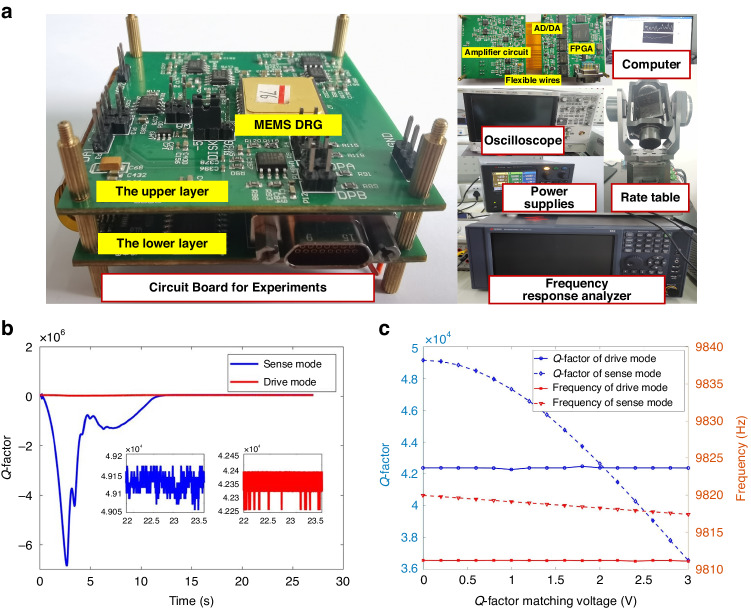
Experimental setup and open-loop Q-factor test results. **a** Experimental verification platform. **b** Experimental results of Q-factor testing. **c** Frequencies and Q-factors for both drive and sense modes

Figure 5a depicts the experimental verification platform for the FPGA-based measurement and control circuit. This platform includes a custom-designed experimental circuit board, aligned with the circuit schematic diagram portrayed in Fig. 4, alongside DC power supplies, an oscilloscope, a signal analyzer, a computer, and a rate turntable. The experimental circuit board has a dual-layer structure, with the upper layer serving as the installation site for the MEMS DRG and the analog amplifier circuitry layout. The lower layer predominantly houses an AD/DA conversion circuit and digital hardware circuitry centered on the FPGA chip. Signal transmission between these upper and lower layers is achieved through flexible wiring. The ±24 V voltage from the DC power supplies is directed to the experimental circuit board via the interface on the lower layer. The oscilloscope facilitates the observation of input and output signals from both drive and sense modes. The use of a signal analyzer allows key characteristic parameter testing of the MEMS DRG. The rate turntable contributes to the calibration of the scale factor. Data acquisition and analysis were conducted on a computer at a sampling frequency of 2 kHz.

### Open-loop Q-factor matching experiment

The Q-factor serves as a metric for characterizing the energy dissipation of a MEMS gyroscope by exerting a direct influence on performance parameters such as sensitivity and resolution. Traditional methods for evaluating the Q-factor primarily include attenuation and half-frequency methods. However, considering their inability to function during gyroscope operation, these methods cannot be applied to the proposed automatic Q-factor matching method. Consequently, this study compares the Q-factors derived from the attenuation method with those obtained through the newly introduced online test method. After powering the experimental circuit board, all the loops (except for the Q-factor matching loop) initiate their operations. In cases where damping is not matched, the tuning voltage stabilizes at 9.71 V. Employing a signal analyzer for a frequency sweep test on both drive and sense modes yields a frequency split of less than 0.06 Hz, indicating that the MEMS DRG is in the mode-matching state. The experimental results illustrating the Q-factor testing are depicted in Fig. 5b, with a Q-factor of 42,374 observed in the drive mode and 49,147 in the sense mode. Table 2 also presents a comparison between the results obtained by the attenuation method and the online method. The online measurement of the Q-factor aligns closely with that derived from the attenuation method, exhibiting a measurement error below 4%.

To ascertain the actual impact of the resistive damper on gyroscope mode damping and stiffness, an open-loop damping adjustment experiment is conducted. Following the activation of the MEMS DRG, the Q-factor matching voltage *V*_*Qy*_ is applied to the resistive damper of the sense mode in an open-loop manner, and *R*_*Qx*_ = 50 kΩ is used. The changes in the resonant frequency and Q-factor for both the drive and sense modes are tested and recorded for varying *V*_*Qy*_ values, as demonstrated in Fig. 5c. The experimental findings reveal that *V*_*Qy*_ exerts a negligible influence on the Q-factor and frequency of the drive mode when applied to the sense mode. Remarkably, the *V*_*Qy*_ values showcase similar frequency tuning capabilities to those of the tuning voltage, with a voltage of 3 V leading to a reduction in the sense mode frequency from 9819.96 Hz to 9817.41 Hz. Therefore, a voltage of 2.05 V effectively achieves complete Q-factor matching for the MEMS DRG.

### Automatic Q-factor matching experiment

**Fig. 6:**
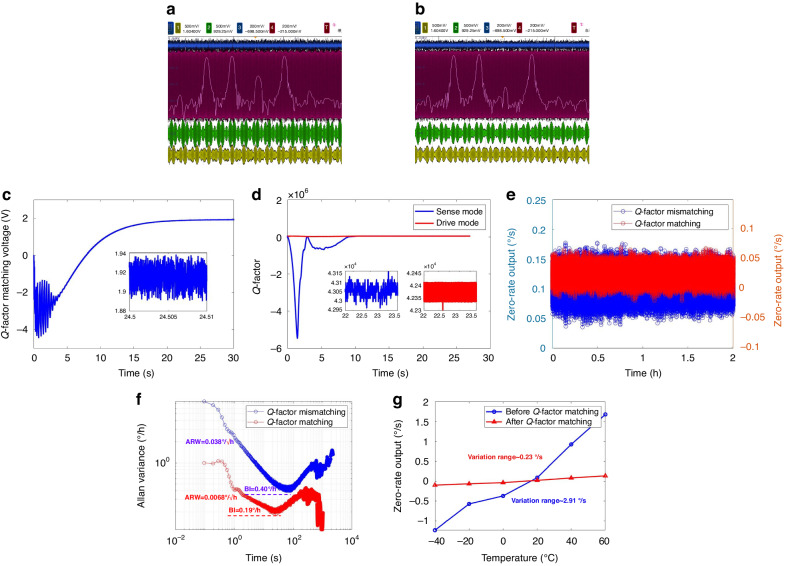
Experimental results of the automatic Q-factor matching method. **a** Input and output signals before Q-factor matching. **b** Input and output signals after Q-factor matching. **c** Q-factor matching voltage. **d** Q-factors of the drive mode and sense mode. **e** Zero-rate output. **f** Allan variance analysis results. **g** ZRO of the MEMS DRG before and after Q-factor matching at different temperatures

Figure 6a, b shows the changes in the input and output signals of the drive and sense modes, as observed by the oscilloscope, before and after activating the Q-factor matching loop. The drive mode’s input signal is represented by the blue signal, while its output signal is represented by the red signal. Similarly, the green signal represents the input signal of the sense mode, and the yellow signal represents its output signal. Additionally, the pink curve corresponds to the FFT curve of the sense mode’s output signal. The output of the sense mode consists of the response amplitudes of four distinct frequency signals. From left to right, these signals represent an auxiliary signal for online Q-factor testing with a 0.6 V amplitude and a frequency difference of 60 Hz from the drive mode, a double-sideband signal composed of the upper band signal and the lower band signal for mode matching with a 0.8 V amplitude and a ±30 Hz frequency difference from the drive mode, and finally, the in-phase error signal of the MEMS DRG. The frequency intervals of 60 Hz and ±30 Hz are chosen based on comprehensive consideration of the experimental results. This selection ensures that the demodulated control signals required by each control loop in the sense mode have a sufficient signal-to-noise ratio while also avoiding mutual coupling interference between the control loops due to proximity to the resonance frequency of the MEMS gyroscope.

The experimental results demonstrate that upon initiating the Q-factor matching loop, there is a noticeable decrease in the amplitude of the in-phase error signal within the FFT analysis. This reduction highlights the significant impact of the Q-factor matching loop on the elimination of the ZRO in the MEMS DRG.

Upon initiating the Q-factor matching loop, the PI controller automatically generates a Q-factor matching voltage, as shown in Fig. 6c. The Q-factor matching voltage stabilizes at 1.92 V, resulting in a reduction in the tuning voltage from 9.71 V to 8.53 V. Simultaneously, the Q-factors of the drive mode and sense mode are displayed in Fig. 6d. The Q-factor of the sense mode decreases from 49,147 to 43,211, while the Q-factor of the drive mode remains relatively unchanged at 42,374. These experimental findings demonstrate that the automatic Q-factor matching method effectively reduces the Q-factor mismatches of the MEMS DRG by 87%.

To validate the effectiveness of the Q-factor matching method in enhancing the performance of the MEMS DRG, a calibration process is conducted by placing the experimental circuit board on a rate turntable. The resulting scale factor is measured to be 78.53 mV°/s. Subsequently, the ZRO were collected for a duration of 1.5 h under two conditions: Q-factor mismatching and Q-factor matching. These responses are then analyzed based on Allan variance.

Figure 6e, f displays the ZRO and Allan variance analysis before and after Q-factor matching. Notably, the ZRO of the MEMS DRG decreased from 0.11°/s to 0.025°/s, indicating a significant improvement in the bias reduction. Moreover, there is a discernible enhancement in noise levels.

The results obtained from the Allan variance analysis reveal that the bias instability (BI) of the MEMS DRG decreased from 0.40°/h to 0.19°/h, demonstrating an improvement of a factor of 2.1. Additionally, the angle random walk (ARW) decreased from 0.038°/√h to 0.0068°/√h, representing an increase of 5.6. Based on these experimental findings, it is evident that implementing the automatic Q-factor matching method can significantly enhance the performance of the MEMS DRG.

However, the additional closed-loop control introduces additional control interference, leading to increased noise in the gyroscope rate output. To eliminate the interference signal from the Q-factor matching loop on the force-balanced loop, we adjust the cutoff frequency of the low-pass filter in the force-balanced loop from 20 Hz to 5 Hz after initiating the Q-factor matching loop. At this point, the ARW of the MEMS DRG far exceeds the mechanical thermal noise of the MEMS DRG, indicating that the circuit noise has a dominant effect on the ARW. Therefore, we propose that the observed improvement in the ARW is not due to Q-factor matching. In contrast, reducing the Q-factor would increase the mechanical thermal noise of the MEMS DRG (the theoretical values of the mechanical thermal noise of the MEMS DRG investigated in this paper before and after Q-factor matching are 0.0024°/√h and 0.0025°/√h, respectively).

To verify the tuning effect of the proposed automatic Q-factor matching method at different temperatures, we selected six temperature points (–40 °C, –20 °C, 0 °C, 20 °C, 40 °C, and 60 °C) within the temperature range of –40 °C to 60 °C. We record the values of *Q*_*x*_, *Q*_*y*_, and the Q-factor matching voltage *V*_*Qy*_ obtained through the online test method. According to Table 3, the Q-factor matching voltage *V*_*Qy*_ for the output of the Q-factor matching loop varies slightly at different temperatures. However, Δ*Q* is always controlled within 1000, indicating that the proposed automatic Q-factor matching loop can achieve effective matching of the Q-factor even under variable temperature conditions.

**Table 3:** *Q*_*x*_, *Q*_*y*_ and *V*_*Qy*_ at different temperatures

Temperature (°C)	*Q*_*x*_	*Q*_*y*_	Δ*Q*	Q-factor matching voltage *V*_*Qy*_ (V)
–40	48,452	49,205	753	1.80
–20	46,101	46,728	627	2.09
0	44,303	45,087	784	1.73
20	42,525	43,317	792	1.94
40	40,577	41,422	845	1.82
60	38,964	39,877	913	1.43

Moreover, the ZRO of the MEMS DRG before and after Q-factor matching at different temperatures are recorded, as shown in Fig. 6g. Without Q-factor matching, the ZRO of the MEMS DRG changes from –1.23°/s to 1.68°/s, with a variation of 2.91°/s within the temperature range of –40 °C to 60 °C. After Q-factor matching, the ZRO of the MEMS DRG changes from –0.09°/s to 0.14°/s, with a variation of 0.23°/s within the same temperature range. These experimental results demonstrate that dynamic real-time Q-factor matching can effectively reduce the ZRO caused by the damping coupling error and decrease the temperature sensitivity of the ZRO, enabling the MEMS DRG to maintain reliable performance in changing environments.

## Discussion

The proposed automatic Q-factor matching method is verified to eliminate error signals that are in phase with the Coriolis force signal, coupled to the sense mode, and caused by the Q-factor mismatches in the resonant structure of the MEMS vibration gyroscope. By reducing the ZRO, the proposed method enhances the performance of the MEMS DRG. In this study, the MEMS DRG is utilized as an experimental object, and online identification of the Q-factor mismatches is accomplished using a novel Q-factor online test method. Additionally, automatic matching of the Q-factors for both drive and sense modes is achieved by adjusting the gyroscope mode damping coefficient through the resistive damper and the automatic control principle, enabling successful automatic Q-factor matching. The experimental results demonstrate that the Q-factor identification error in gyroscope mode exceeded 4%, and 87% of the Q-factor mismatches were eliminated through automatic Q-factor matching. Furthermore, the ZRO of the MEMS DRG decreased from 0.11°/s to 0.025°/s, resulting in improved noise levels within the ZRO. The BI of the MEMS DRG decreased from 0.40°/h to 0.19°/h, which is an increase of 2.1 times. Notably, the achievement of a smaller tuning error and the minimization of phase errors in the hardware circuit are crucial for enhancing the precision of Q-factor matching, as these factors directly influence the Q-factor test error in the sense mode. This method also is readily applicable to other MEMS vibration gyroscopes with degenerate drive and sense modes.

## Materials and methods

The multiring structure of the MEMS DRG is composed of 40 concentric rings. The outermost ring has a radius of 2275 μm, while the innermost ring has a radius of 1680 μm. Each ring has a width of 10 μm, and the spokes separating the rings have a width of 5 μm. The fabrication of the MEMS DRG utilizes the silicon-on-glass (SOI) process, and it is packaged using vacuum packaging technology. The automatic mode-matching method discussed in this paper is based on the principle of response amplitude detection of a doubled-sideband signal, as described in detail in ref. ^32^. For the resistive damper, the capacitor has an initial capacitance (*C*_0_) of 0.21 pF, and the initial spacing (*d*_0_) between the plates is 2.5 μm. In terms of the experimental setup, the circuit board incorporates AD8642 and AD8221 to design the differential C/V amplifier circuit. The digital converter utilized is the 24-bit LTC2380-24, and the digital-to-analog converter is the 16-bit AD5541A.
